# Ultrasonic Texture Analysis for Predicting Acute Myocardial Infarction

**DOI:** 10.1016/j.jcmg.2025.06.018

**Published:** 2025-08-15

**Authors:** Ankush D. Jamthikar, Quincy A. Hathaway, Kameswari Maganti, Yasmin Hamirani, Sabahat Bokhari, Naveena Yanamala, Partho P. Sengupta

**Affiliations:** a Division of Cardiovascular Diseases and Hypertension, Department of Medicine, Rutgers Robert Wood Johnson Medical School, New Brunswick, New Jersey, USA;; b Department of Radiology, University of Pennsylvania, Philadelphia, Pennsylvania, USA.

**Keywords:** apical views, echocardiography, myocardial infarction, radiomics, infarct localization, ultrasomics

## Abstract

**BACKGROUND:**

Acute myocardial infarction (MI) alters cardiomyocyte geometry and architecture, leading to changes in the acoustic properties of the myocardium.

**OBJECTIVES:**

This study examines ultrasomics—a novel cardiac ultrasound-based radiomics technique to extract high-throughput pixel-level information from images—for identifying ultrasonic texture and morphologic changes associated with infarcted myocardium.

**METHODS:**

The authors included 684 participants from multisource data: a) a retrospective single-center matched case-control dataset; b) a prospective multicenter matched clinical trial dataset; and c) an open-source international and multivendor dataset. Handcrafted and deep transfer learning–based ultrasomics features from 2- and 4-chamber echocardiographic views were used to train machine learning (ML) models with the use of leave-one-source-out cross-validation for external validation.

**RESULTS:**

The ML model showed a higher AUC than transfer learning–based deep features in identifying MI (AUC: 0.87 [95% CI: 0.84–0.89] vs AUC: 0.74 [95% CI: 0.70–0.77]; *P* < 0.0001). ML probability was an independent predictor of MI even after adjusting for conventional echocardiographic parameters (adjusted OR: 1.03 [95% CI: 1.01–1.05]; *P* < 0.0001). ML probability showed diagnostic value in differentiating acute MI, even in the presence of myocardial dysfunction (averaged longitudinal strain [LS] <16%) (AUC: 0.84 [95% CI: 0.77–0.89]). In addition, combining averaged LS with ML probability significantly improved predictive performance compared with LS alone (AUC: 0.86 [95% CI: 0.80–0.91] vs AUC: 0.80 [95% CI: 0.72–0.87]; *P* = 0.02). Visualization of ultrasomics features with the use of a Manhattan plot discriminated infarcted and noninfarcted segments (*P* < 0.001) and facilitated parametric visualization of infarcted myocardium.

**CONCLUSIONS:**

This study demonstrates the potential of cardiac ultrasomics to distinguish healthy from infarcted myocardium and highlights the need for validation in diverse populations to define its role and incremental value in myocardial tissue characterization beyond conventional echocardiography.

## CENTRAL ILLUSTRATION

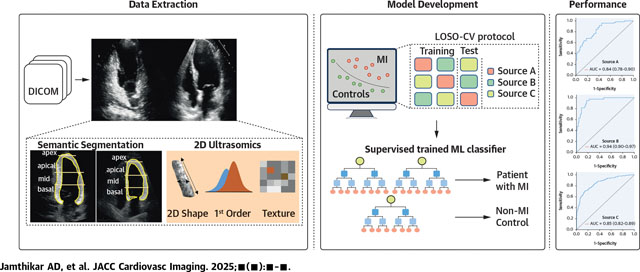

**Ultrasomics for Predicting MI** DICOM files for apical 2- and 4-chamber images are segmented into American Heart Association–defined left ventricular myocardial segments, and radiomic features are extracted across the cardiac cycle; features are standardized, and a machine learning (ML) model is trained and evaluated to predict MI using a leave-one-source-out cross-validation (LOSO-CV) protocol. MI = myocardial infarction.

Acute myocardial infarction (MI) is one of the leading causes of morbidity and mortality globally. The prevalence of the disease approaches 3 million people worldwide, with 605,000 new MIs diagnosed annually in the U.S.^1^ Echocardiography is a rapid, noninvasive, portable, and inexpensive imaging modality, making it the preferred technique for assessing MI patients. Although echocardiography visualizes the effects of ischemia and MI on regional and global myocardial function, direct identification and quantification of infarcted tissue remains challenging and has not been extensively explored, especially compared with cardiac magnetic resonance (CMR) imaging, which is considered to be the criterion standard for infarct tissue characterization.^2^

The recent developments in image analysis and novel informatics approaches have augmented methods that can extract information from medical images that quantify their phenotypic characteristics in an automated high-throughput manner. The application of such pixel-level image analyses is referred to as ‘radiomics,’ which converts images into structured mineable datasets. This data-driven framework “discovers” quantitative information within images by extracting high-dimensional data (“features”) beyond the visually perceptible, with the use of computational statistics (often based on machine learning [ML] algorithms) to predict or establish association with a clinical phenotype or endpoints. Such features have been used recently to help prognosticate and predict treatment outcomes in cancer research and neuroscience.^3^ Previous studies have reported that the intensity of the ultrasound backscatter is related to the physical properties of the myocardium.^4,5^ We have recently described the application of cardiac ultrasound radiomics, also called “ultrasomics,” for predicting cardiac remodeling and delayed gadolinium enhancement CMR-derived fibrosis.^6,7^ Ultrasomics may also carry unique and specific information about post-MI infarct size quantification—an important predictor of mortality and a key endpoint for MI cardioprotection strategies.^8,9^ However, there is limited information regarding the application of ultrasomics for assessing patients presenting with MI.

Previous approaches using conventional ML and deep learning for diagnosing MI have typically relied on echocardiographic frames or videos encompassing the entire cardiac chambers or endocardial border motion to detect segmental wall motion abnormalities.^10–12^ However, this method lacks specificity for identifying acute MI. The present investigation had 3 objectives: 1) to compare the diagnostic value of handcrafted ultrasomics (extracted from echocardiography images using predefined mathematical algorithms) with deep learning–derived features (generated through automated methods such as convolutional neural networks) for distinguishing patients with and without acute MI; 2) to explore the independent and incremental value of ultrasomics over conventional echocardiographic parameters including longitudinal strain (LS) for detecting acute MI; and 3) to assess the feasibility of ultrasomics features to localize infarcted tissue and create a parametric map of infarcted myocardium with the use of paired CMR assessments.

## METHODS

### CLINICAL POPULATION AND STUDY DESIGN.

**FIGURE 1:**
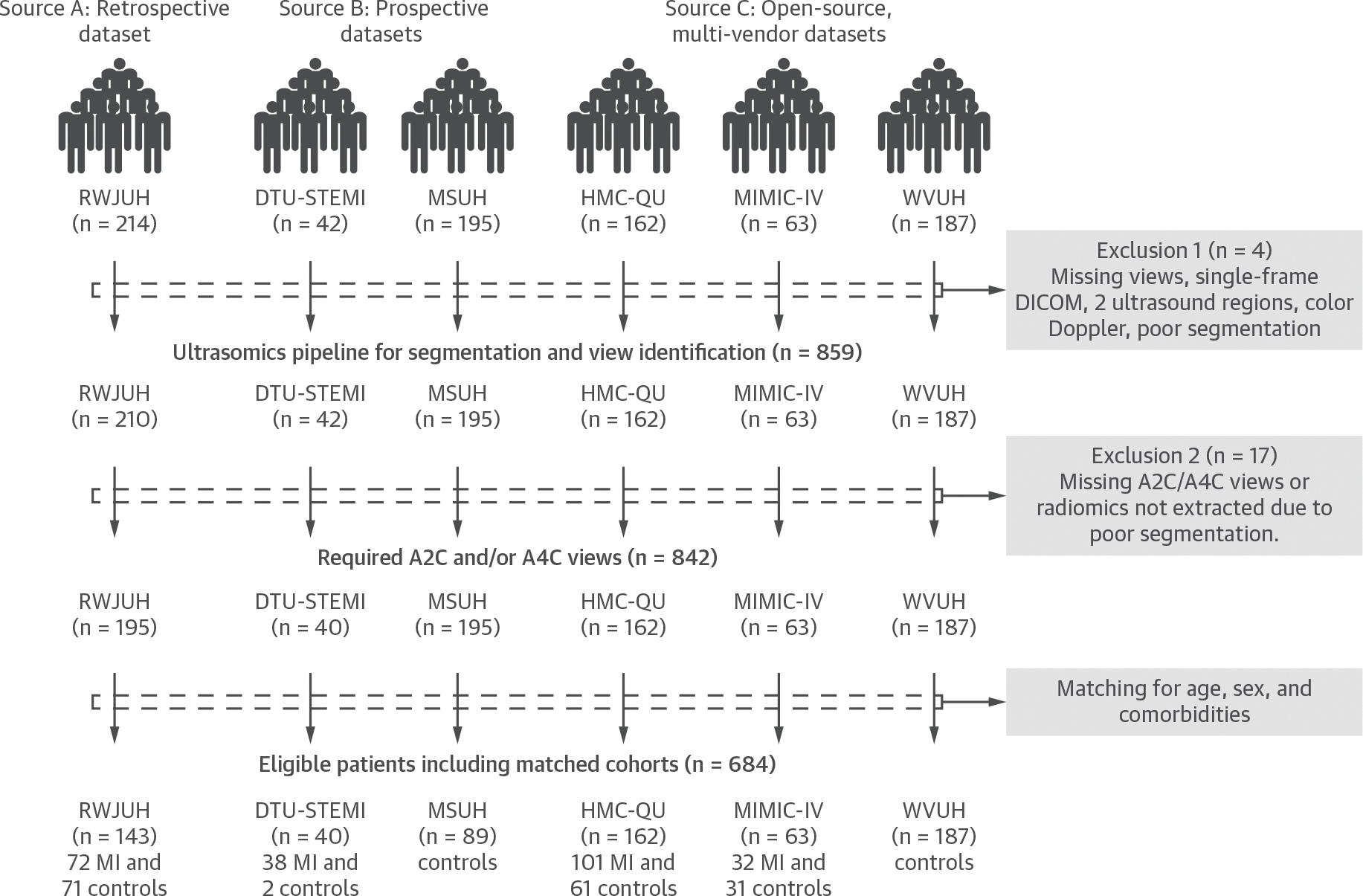
The CONSORT Diagram Participants from 6 centers, grouped into 3 data sources, underwent segmentation and view identification; after matching for age, sex, and comorbidities, 684 participants were eligible. WVUH = West Virginia University Hospital; RWJUH = Robert Wood Johnson University Hospital; DTU-STEMI = Door-To-Unload in STEMI; MSUH = Mount Sinai University Hospital. A2C = apical 2-chamber; A4C = apical 4-chamber.

A total of 684 subjects from 6 sites were divided into 3 data sources: A) retrospective single-center matched case control subjects (data source A), B) a prospective multicenter matched clinical trial dataset (data source B), and C) an open-source unmatched international and multivendor dataset (data source C) (Figure 1).^10,13–17^

The retrospective single-center matched case-control cohort (data source A) comprised 143 subjects (72 MI and 71 non-MI control subjects) admitted from January 2023 to December 2024 at Robert Wood Johnson University Hospital (RWJUH) (New Brunswick, New Jersey, USA) and matched for age, sex, and underlying comorbidities (Supplemental Methods, Section I).

The prospective clinical trial dataset (data source B) comprised 40 participants from the DTU-STEMI (Door-To-Unload in STEMI; NCT03000270) trial—a prospective, multicenter, randomized pilot trial involving 14 centers in the U.S.^13^—and 89 matched non-MI control subjects enrolled from March 2013 to December 2015 from the Mount Sinai University Hospital (MSUH) (New York, New York, USA) in a clinical study where subjects underwent echocardiography and computed tomographic coronary angiography for exploring the development of ML models of diastolic dysfunction from surface electrocardiography (ECG).^15^

The open-source international dataset (data source C) comprised 162 individuals (101 MI cases, 61 non-MI control subjects) collected from 2018 to 2019 from Hamad Medical College–Qatar University (HMC-QU) (Doha, Qatar),^10,12^ and 32 participants with MI from the MIMIC-IV-ECHO (Medical Information Mart for Intensive Care IV With Echocardiogram), who were admitted from 2017 to 2019.^17^ This dataset was enriched with control subjects with varying age and risk exposures to understand how ultrasomics features associate with MI independently from age and risk factors. This included 31 non-MI older control subjects and a multivendor database (Supplemental Table 1) comprising 187 non-MI control subjects (57 healthy and 130 with cardiovascular risk factors) collected from July 2017 to February 2018 from West Virginia University Hospital (WVUH) (Morgantown, West Virginia, USA).^14^

All databases were approved by the local institutional review boards.^10,13,16,18^ Participants in the prospective cohorts provided written informed consent. The studies adhered to institutional and national ethical standards and the 1964 Helsinki Declaration.

### DEFINING THE STATUS OF MYOCARDIAL INFARCTION.

#### Data source A.

Patients with ST-segment elevation myocardial infarction (STEMI) in the RWJUH dataset were identified with the use of clinical findings, electrocardiographic changes, and biomarker levels according to the current universal definition of MI (Supplemental Methods, Section II).^19^

#### Data source B.

In the DTU-STEMI trial,^13^ patients underwent CMR imaging on days 3 to 5 and day 30 (±7 days) using standard protocol.^13^ A central core laboratory (Duke Cardiovascular Magnetic Resonance Center, Durham, North Carolina, USA) assessed deidentified images. The presence of an infarct in each of the 17 myocardial AHA (American Heart Association)–defined echocardiography segments was labeled based on CMR-defined delayed hyperenhancement or late gadolinium enhancement. In addition, we considered delayed enhancement scores determined according to the well established 5-point grading system outlined by the AHA,^20^ where 0 indicates no hyperenhancement, 1 represents 1%–25%, 2 signifies 26%–50%, 3 corresponds to 51%–75%, and 4 reflects 76%–100% involvement.^20^ The CMR delayed enhancement scores were used to define segment-level infarcts (hyperenhancement score >0).

#### Data source C.

In the HMC-QU database, patients with STEMI were admitted and treated with coronary angiography or angioplasty, with echocardiography performed within 24 hours or before intervention. Non-MI subjects were evaluated for other clinical reasons. Each myocardial segment was labeled as infarct related or normal based on regional wall motion abnormalities. Cardiac cycle frames (end-diastole and end-systole) were defined from ECG data or by identifying frames with the largest and smallest left ventricular (LV) areas when ECG was unavailable.^10,12,21^ In the MIMIC-IV-ECHO database, STEMI patients were identified by ICD-10 codes (I2111, I2119, I2102, I213, I2109, I2121). Control patients were selected with the ICD-10 code I10, with normal echocardiographic measurements.^22^

### ECHOCARDIOGRAPHY IMAGE ANALYSIS AND SEGMENTATION.

The details on echocardiography and image settings are provided in Supplemental Table 1. All comprehensive 2-dimensional (2D) and Doppler transthoracic echocardiographic images were acquired by expert sonographers per guidelines.^23^ Average LS was measured from apical 2-chamber (a2c)- and 4-chamber (a4c) views with the use of validated software.^22^ Our automated echocardiography imaging workflow for ultrasomics comprises 4 stages: preprocessing, view identification, segmentation, and ultrasomics feature extraction. The preprocessing stage converted 2D echocardiograms with varying resolutions and frame rates (Supplemental Table 1) from diverse formats (.avi, .mp4) into standard DICOM format with the use of Sante DICOM software (version 7.9.4, 64-bit) for harmonization. DICOM files with Doppler data or dual ultrasound regions were excluded. The view identification stage classified these processed DICOM files to identify a2c and a4c transthoracic echocardiography views.

During segmentation, the LV myocardium was delineated in each frame throughout a cardiac cycle (end-diastole to end-systole). A black-and-white (ie, binary) mask marked the myocardial region within the echocardiographic image for both apical views. To ensure uniformity and harmonization in ultrasomics extraction, grayscale LV myocardium images and binary masks were saved at a standardized resolution of 1,024 × 1,024 pixels for all databases (Supplemental Table 1).

Using an automated algorithm, we subsequently delineated the LV segments from the segmented myocardium as per the conventional AHA-defined myocardial segments for the a2c and a4c views. The apical segment (apical cap, segment 17) was excluded from the segmental analysis per guideline recommendations.^24^ Spatial resolution was maintained during segmentation to ensure each segment retains its size and relative location within the LV myocardium. The algorithm uses LV myocardial binary masks to define segments, ensuring consistent segmental positioning across all frames.

The original image and its corresponding binary myocardial segments were then fed to an ultrasomics pipeline to extract 2D shape-based, first-order, and texture-based features. Ultrasomics feature extraction targets 2D features from static images, not directly capturing segmental motion (eg, wall motion abnormalities). Instead, an average of these static features across all frames represents the temporal and spectral characteristics of the cardiac cycle, providing a broader view of segmental rhythm changes. The complete automated pipeline was developed in open-source Python (version 3.7, Python Software Foundation) and has been previously validated for extracting ultrasomics features.^6,16^

### MORPHOLOGY AND TEXTURE-DRIVEN HANDCRAFTED ULTRASOMICS.

PyRadiomics (version 3.0.1, Python Software Foundation^25^) and SimpleITK (version 2.2.0, Insight Software Consortium^26^) used within the open-source Python framework facilitated the extraction of ultrasomics features, also referred to as handcrafted radiomics (HCR). To maintain consistency, we extracted ultrasomics features from 12 segments across all databases, excluding the apical cap because it lacks inward segmental motion activity and is therefore not recommended in guidelines for segmental wall assessments.^10,24^ The details of these HCR features that were used for ML model development are provided in Supplemental Methods, Section III.

### DEEP LEARNING–BASED ULTRASOMICS.

Similarly to ultrasomics, to evaluate deep transfer*–l*earning radiomics (DTL) features, we compared 3 3-dimensional (3D) convolutional neural network (CNN)–based architectures: Slow-Fast R50,^27^ a channel-separated convolutional network,^28^ and a transformer-based model. Using transfer learning, features were captured from the final classification layer of these 3D-CNN architectures. Each myocardial segment video produced 400 DTL features, resulting in 4,800 features from 12 segments across a2c and a4c views per patient. These features were then, used for feature engineering and ML model development. As preprocessing (Supplemental Methods, Section IV), each ultrasomics feature was standardized (mean zero, unit variance).^29^ To address scanner-related variability and reduce batch effects in the radiomics data, we applied the ComBat harmonization technique—a widely used and robust method for correcting batch effects.^30^ The details of harmonization steps are provided in Supplemental Methods, Section V.

### MACHINE LEARNING MODEL DEVELOPMENT.

The ML model (Central Illustration) development was performed through an automated machine learning (AutoML) pipeline (H2O.ai, version 3.44.0.2^31^). The AutoML platform uses a series of ML algorithms,^31^ including generalized linear models, deep neural networks, distributed random forests, gradient boosting machines, and extreme gradient boosting.

Standard hold-out and cross-validation (CV) methods based on random splitting of combined data can be expected to be overoptimistic when deploying models to sources not represented in the dataset. Therefore, to evaluate model performance specifically that includes patients collected across multiple sites, we used a leave-one-source-out cross-validation (LOSO-CV) strategy. Unlike standard k-fold methods that assume datapoints are independent, LOSO-CV keeps all data from the same group together in the training or testing set. This method of external validation has been advocated for assessing the generalizability of model performance across diverse multisite patient populations.^32,33^ We followed our previously published PRIME checklist for the development of ML models.^34^ The prediction probabilities served as a continuous ultrasomics infarction score, with any value ≤0.5 indicating the presence of infarction and <0.5 indicating the absence of infarction.

### PERFORMANCE EVALUATION AND STATISTICAL ANALYSIS.

Baseline clinical characteristics are presented as median (Q1-Q3) for continuous variables and n (%) for categoric variables. Continuous variables across multiple groups were compared by means of a one-way analysis of variance to see if the variable is normally distributed or if the Kruskal-Wallis test is otherwise. Categoric variables were assessed by means of Fisher test for contingency table values <5 or the chi-square test otherwise. Ultrasomics features from infarcted and noninfarcted segments per patient across the cohort were analyzed by means of a paired Mann-Whitney *U*-test, with significant features visualized using a Manhattan plot. A sensitivity analysis assessed the reproducibility and robustness of significant ultrasomics features to noise and altered image gains, simulating diverse scanner settings, using the intraclass correlation coefficient. The performance of ML models was assessed by means of LOSO-CV. Predictions for each source were used to evaluate the area under the receiver operating characteristic curve (AUC), along with other performance metrics such as sensitivity, specificity, F1 score, and accuracy. The overall performance across all 3 data sources was also calculated and reported as their 95% CI, estimated with the use of a bootstrapping technique. An overall ROC curve was also generated by concatenating the predictions from each held-out set. The prediction probabilities from each held-out set during the CV process were used to create this curve, providing a comprehensive evaluation of the model’s performance.^35^ The difference in AUCs between groups was estimated by means of the DeLong test. Univariate and multivariate logistic regression analyses were performed in the entire cohort using ML model predictions from each held-out group and echocardiographic features to predict MI. Variables with *P* < 0.05 in univariate analysis were included in the multivariate regression model. All analyses were performed with the use of Python (version 3.7) and MedCalc (version 12.5.0.0), with *P* < 0.05 considered to be significant.

### REPRODUCIBILITY OF ULTRASOMICS AND FEASIBILITY ANALYSIS.

We identified ultrasomics features that significantly differentiate infarcted from normal myocardium with the use of paired Student’s *t*-tests and Manhattan plots. Reproducibility analysis was conducted to evaluate variability in these features due to device settings and image quality. Unlike our previous studies, which used still frames from the parasternal long-axis view,^7^ the present approach used ultrasomics from the cardiac cycle in apical views, allowing us to capture static and dynamic features, including temporal and spectral variations across the cardiac cycle. We further tested the robustness of these features under real-world conditions by applying 5 levels of gain adjustments (I[x] + 20, + 40, + 60, + 80, + 100) and gaussian noise (mean = 0; variances 0.01 to 0.05) to images from 10 patients, focusing on texture features from end-diastolic LV segments across 12 segments in a2c and a4c views. To further assess the feasibility of the ultrasomics ML model, we tested the model using input features perturbed by gaussian noise with varying levels of variance (0.01, 0.03, and 0.05). We recorded the overall performance variations from the baseline optimal ML model.

## RESULTS

### BASELINE CHARACTERISTICS.

Tables 1–3 summarize the available baseline characteristics of the participants across data sources A-C, grouped according to the LOSO-CV approach used in the ML model. The participants in the open-source training dataset have been previously published.^10,12,21^

### PREDICTION OF MI BY MEANS OF HCR FEATURES.

**FIGURE 2:**
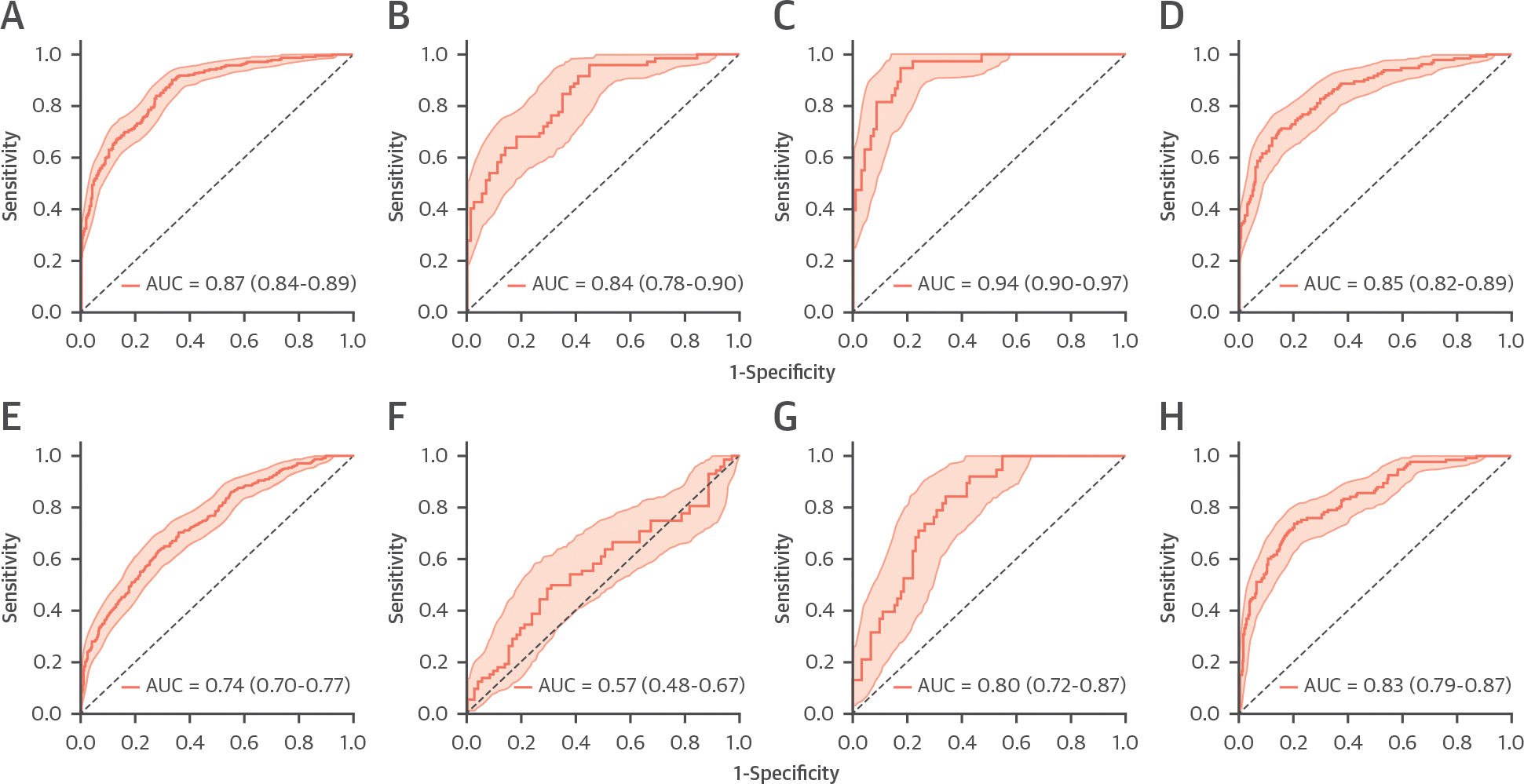
Patient-Level ML Model Performance for MI Prediction ROC curves for patient-level MI prediction using radiomics features: (A to D) handcrafted radiomics and (E to H) deep transfer learning radiomics. Panels A and E show results for the pooled cohort (n = 684); panels B and F for data source A (n = 143); panels C and G for data source B (n = 129); and panels D and H for data source C (n = 412). All analyses were conducted using a leave-one-source-out evaluation. AUC values quantify model discrimination. MI = myocardial infarction; ML = machine learning.

The gradient boost model (XGBoost) emerged as an optimal model for MI prediction, with hyperparameters as listed in Supplemental Table 2. HCR features extracted from frames across the entire cardiac cycle yielded an overall sensitivity of 90.1% (95% CI: 86.4%–93.7%), specificity of 66.4% (95% CI: 62.3%–70.7%), F1-score of 71.8% (95% CI: 67.9%–75.7%), accuracy of 74.9% (95% CI: 71.9%–78.1%), and AUC of 0.87 (95% CI: 0.84–0.89), based on LOSO-CV across all the 3 held-out sets (Table 4). Independent data source–wise performance metrics are presented in Table 4. Figures 2A to 2D present the independent ROC curves for the 3 data sources, along with the overall ROC curve representing the comprehensive performance of the model.

### PREDICTION OF MI BY MEANS OF DTL FEATURES.

Similarly, among the 3 3D-CNN models, the slow-fast ResNet50-based model provided better performance (Supplemental Table 3). The ML model with DTL features extracted from this 3D-CNN yielded an overall sensitivity of 63.4% (95% CI: 57.2%–69.4%), specificity of 71.4% (95% CI: 67.3%–75.6%), F1-score of 58.9% (95% CI: 54.0%–63.9%), accuracy of 68.6% (95% CI: 65.3%–71.9%), and AUC of 0.74 (95% CI: 0.70–0.77) (Table 4). Figures 2E to 2H present the independent ROC curves for the 3 data sources, along with the overall ROC curve representing the comprehensive performance of the DTL-based ML model.

### MODEL PREDICTION ADJUSTED FOR ECHOCARDIOGRAPHY VARIABLES.

Significant univariate echocardiographic predictors of MI in the entire cohort are presented in Table 5. ML prediction probability was concatenated from each independent held-out set to obtain the results for the whole population and compared with other echocardiographic measurements. LV mass index, end-systolic volume, ejection fraction, ratio of early diastolic mitral inflow velocity to early diastolic mitral annulus velocity, wall motion score index, average LS, and ML probability were significant univariate predictors of MI (*P* < 0.05). However, on multivariable regression, average LS (adjusted OR: 1.52 [95% CI: 1.27–1.82]; *P* < 0.0001) and ML probability (adjusted OR: 1.03 [95% CI: 1.01–1.05]; *P* < 0.0001) were independent predictors of MI.

### INCREMENTAL VALUE OF THE ULTRASOMICS ML MODEL.

**FIGURE 3:**
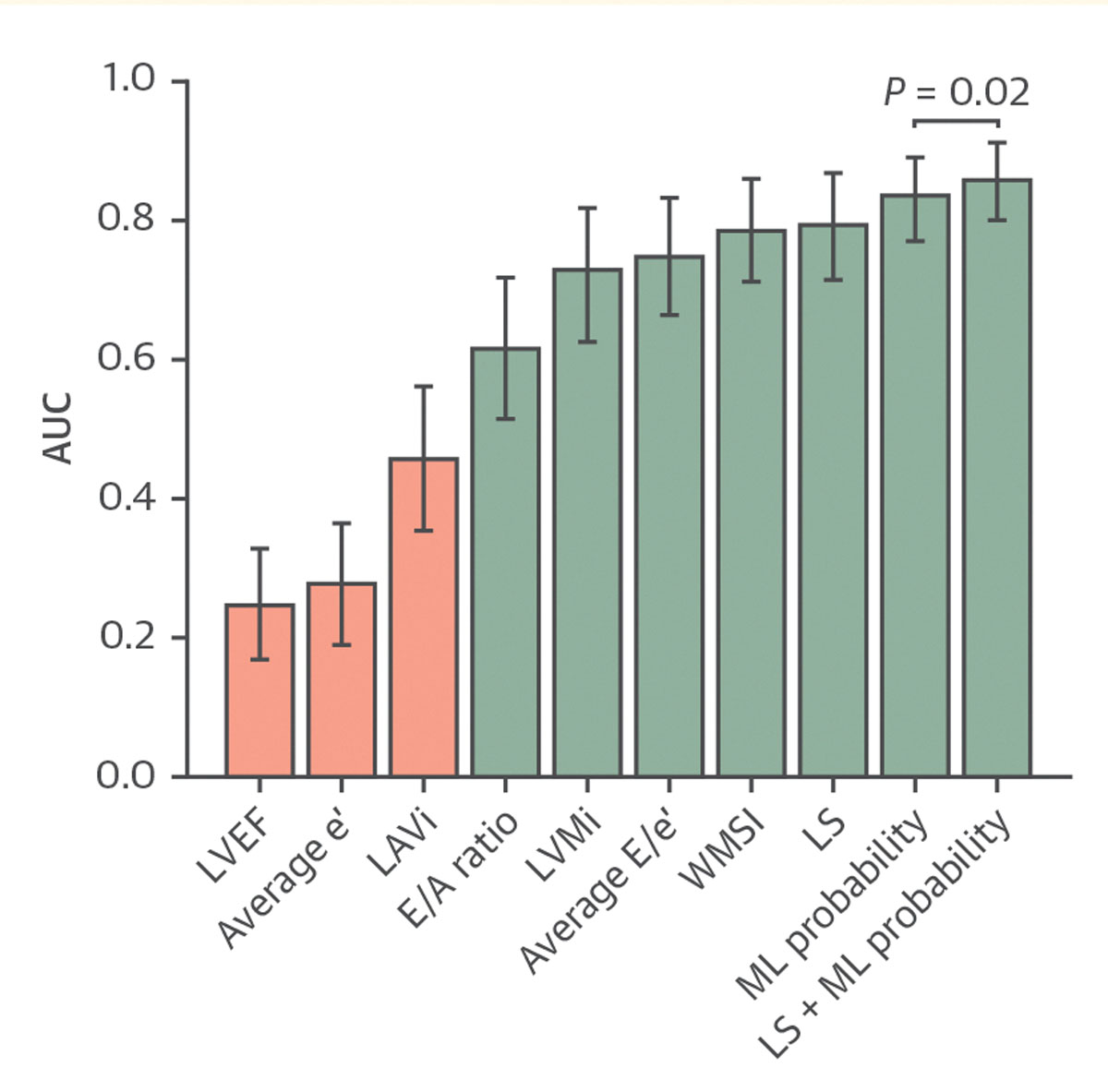
Incremental Value of the Ultrasomics ML Model Over Echocardiographic Measurements The y-axis shows the AUC, and the x-axis lists echocardiographic parameters alongside the ML prediction probability. Bar colors indicate performance: red for AUC < 0.5 and green for AUC ≤ 0.5. A = late diastolic transmitral flow velocity; E = early diastolic transmitral flow velocity; e ′ = early diastolic relaxation velocity at the septal mitral annular position; LS = longitudinal strain; LVEF = left ventricular ejection fraction; LAVi = left atrial volume index; LVMi = left ventricular mass index; ML = machine learning; WMSI = wall motion score index.

To explore whether the ML model could help discriminate the presence or absence of MI in patients with an abnormal echocardiogram, we assessed the incremental value of the ML model for patients with myocardial dysfunction (LS <16%). ML showed an AUC of 0.84 (95% CI: 0.77–0.89). A comparison with other conventional echocardiographic parameters is shown in Figure 3. Besides outperforming other echocardiographic biomarkers, a combination of LS and ML probability further improved the prediction over LS alone (AUC: 0.86 [95% CI: 0.80–0.91] vs AUC: 0.80 [95% CI: 0.72–0.87]; *P* = 0.02).

### ULTRASOMICS SIGNATURES OF INFARCTED MYOCARDIUM.

**FIGURE 4:**
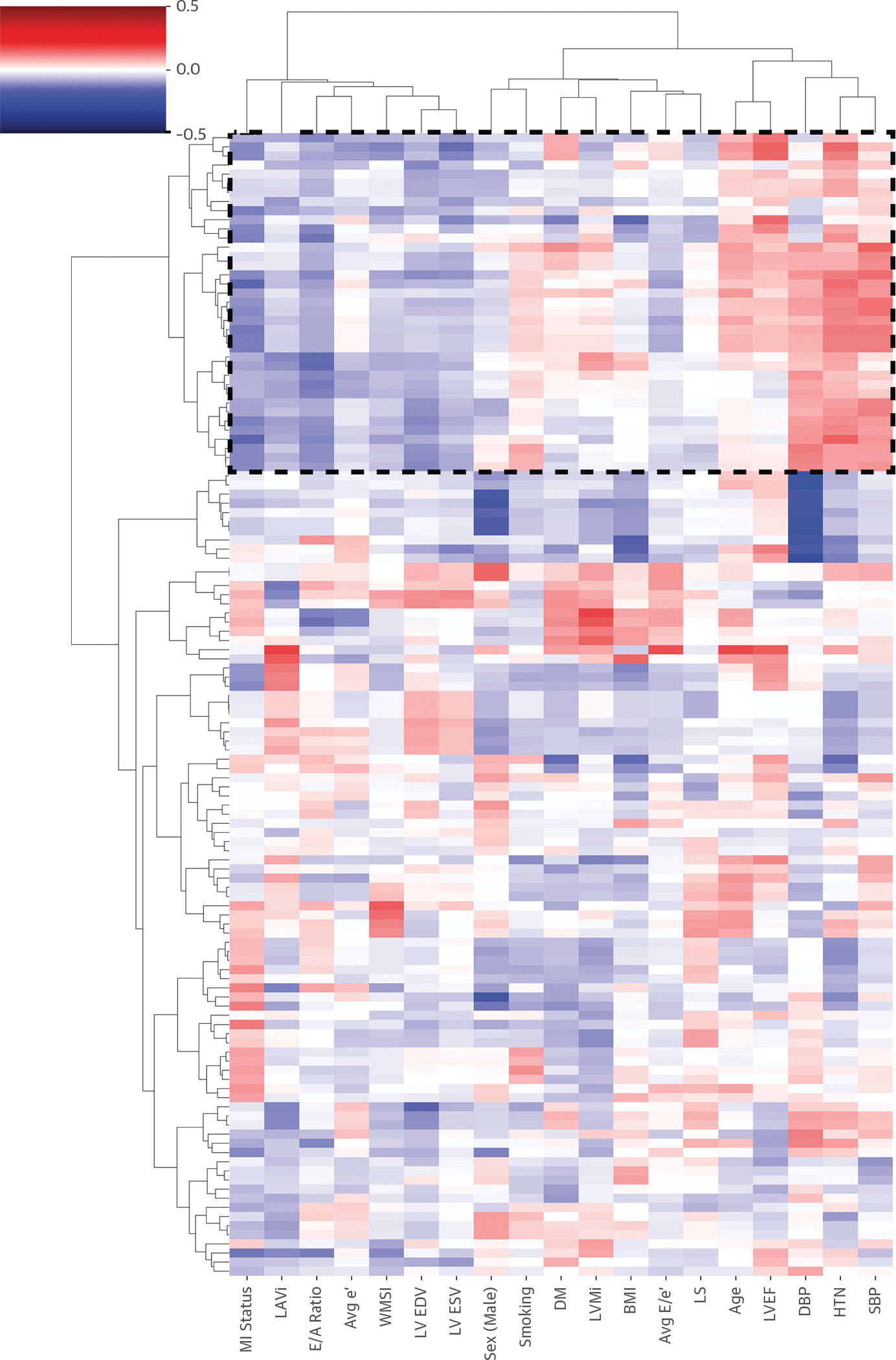
Hierarchical Clustering of Ultrasomics Features Hierarchical clustering heatmap of radiomic features (rows) vs demographic and clinical variables (columns), highlighting grouping patterns and feature-variable relationships. Several ultrasomics features uniquely correlate with myocardial infarction (MI), distinctly from those associated with age and traditional risk factors. BMI = body mass index; DBP = diastolic blood pressure; DM = diabetes mellitus; HTN = hypertension; LS = longitudinal strain; LVEDV = left ventricular end-diastolic volume; LVESV = left ventricular end-systolic volume; SBP = systolic blood pressure; other abbreviations as in Figure 3.

To understand whether the ultrasomics features that distinguish infarcted myocardium are unique from those that are associated with age-, sex-, and risk factor–related changes, we performed an exploratory analysis using agglomerative hierarchical clustering (Figure 4) on data from source C (n = 250). Clustering revealed distinct ultrasonographic patterns: MI cases exhibited unique ultrasomics signatures that were clustered separately from those related to age and risk factors (eg, diabetes, hypertension). Analyzing the feature of importance for the XGboost model developed with the use of LOSO-CV revealed that 8 of the top 20 features identified in the features of importance analysis belong to the highlighted cluster shown in Figure 4 and were unique for identifying MI for non-MI control subjects.

### PARAMETRIC VISUALIZATION OF INFARCTED MYOCARDIUM.

**FIGURE 5:**
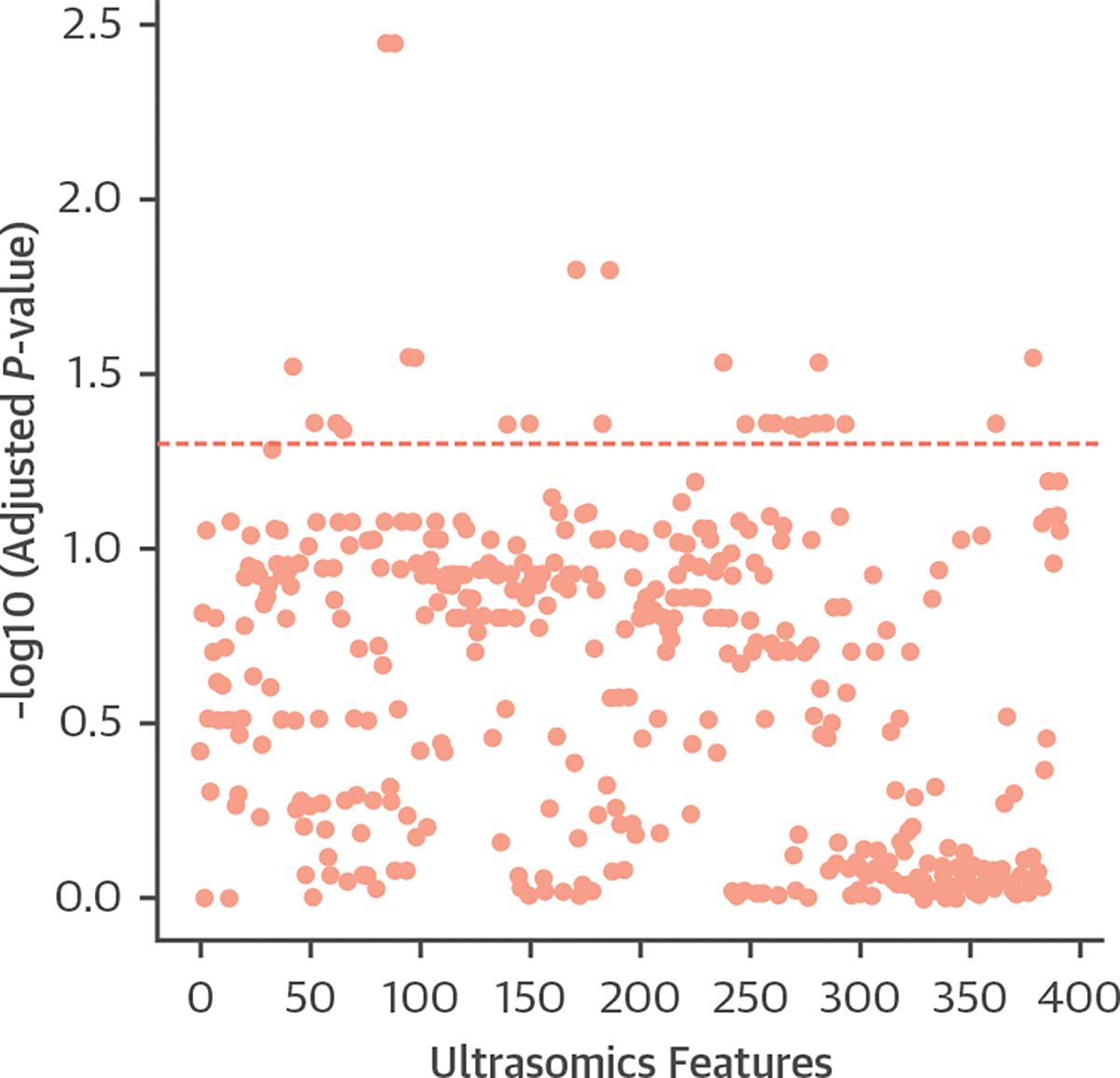
Association of Ultrasomics Features With Segmental Myocardial Infarction Manhattan plot^6^ illustrating the comparison of ultrasomics features for distinguishing infarcted from noninfarcted segmental myocardium. Points represent features, with the y-axis displaying adjusted *P* values (false discovery correction).

**FIGURE 6:**
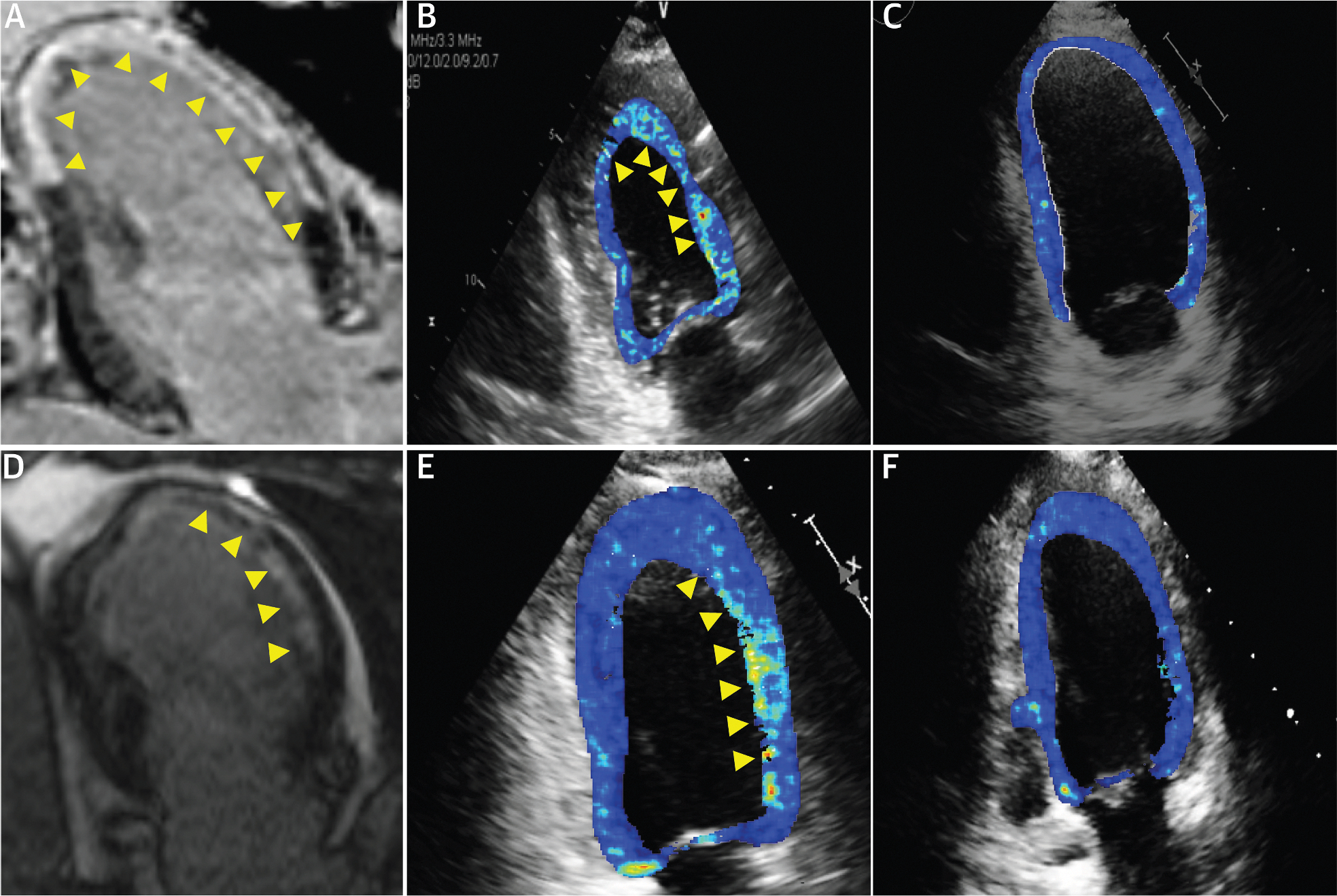
Ultrasomic Texture Feature Maps Over the Left Ventricular Myocardium Patient 1 (A, B) and patient 2 (D, E): paired cardiac magnetic resonance (CMR) and 2-chamber echocardiographic views. (A, D) Arrowheads indicate infarcted myocardium on late gadolinium enhanced images. (B, E) Arrowheads indicate the same regions as identified by the corresponding radiomics feature. (C, F) Control subjects without infarction.

CMR segmental hyperenhancement data from the 40 STEMI patients were used to distinguish infarcted and noninfarcted segments. Ultrasomics features averaged for infarcted and noninfarcted segments in each individual are presented in a Manhattan plot (Figure 5). Among a series of texture features identified, we iteratively displayed the features on the cardiac ultrasound images. We identified the gray-level nonuniformity feature extracted from the gray-level dependence matrix. This feature discriminated between infarcted and noninfarcted segments (*P* = 0.0007) (Supplemental Table 4) and enabled differentiation of patients with and without MI (Figure 6).

### HARMONIZATION AND REPRODUCIBILITY ANALYSIS.

**FIGURE 7:**
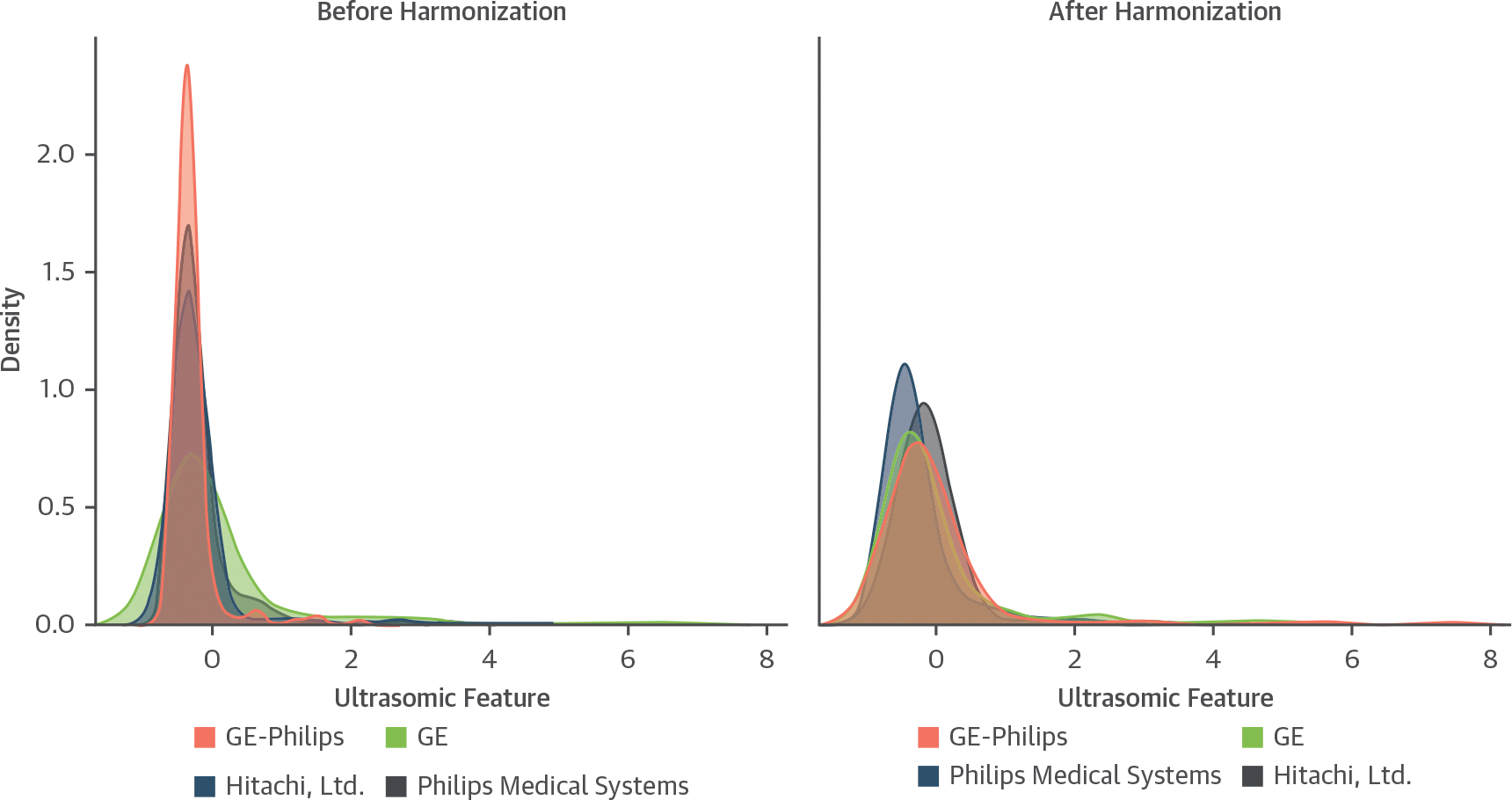
Comparison of Ultrasomic Feature Distributions Before and After Harmonization (Left) Distributions before harmonization, where distinct peaks are observed for each vendor: GE-Philips (red), GE (blue), Hitachi (green), and Philips Medical Systems (purple), indicating scanner-related variability. (Right) Distributions after harmonization, where the distributions are more aligned, indicating reduced scanner-related variability.

The effect of ComBat harmonization in reducing vendor-specific biases in ultrasomics feature distribution is shown in Figure 7 and Supplemental Figure 1. This correction enhanced model performance substantially, with the AUC increasing from 0.74 (95% CI: 0.70–0.78) to 0.87 (95% CI: 0.84–0.89). In previous studies, we confirmed the stability of ultrasomics markers despite image quality variability.^7^ Similarly, in the present study, we analyzed apical views (Supplemental Figures 2 and 3) and tested the impact of gain and gaussian noise. Ultrasomics features from static and dynamic image loops showed high consistency, with an intraclass correlation coefficient of 0.98 (*P* < 0.0001). The feasibility analysis for the ML model demonstrated stability under noise, with minimal AUC variation (<5%) (Supplemental Table 5).

## DISCUSSION

CMR is currently the criterion standard for infarct size quantification in clinical trials,^2,36^ yet its widespread adoption is hindered by cost and accessibility issues. In the present work, we used cardiac ultrasound—the most commonly used cardiac imaging modality—to develop ultrasomics-derived models for identifying patients with MI. Moreover, we illustrate the feasibility of ultrasomics features in parametric visualization of infarcted myocardium within still echocardiography frames, mirroring CMR-based assessment of infarcted myocardium. These novel early insights underscore the promise of ultrasomics approaches for facilitating cardiac ultrasound–based myocardial infarct tissue characterization.

The structural components of the myocardium affect its acoustic properties.^37^ Infarcted myocardium shows stretched and rearranged necrotic myocytes, surrounded by contracted myocytes, edema, hemorrhage, and inflammatory repair processes.^38^ These changes within the myocardium influence ultrasound signal intensity distributions.^39,40^ Myocardial tissue characterization using ultrasound to differentiate infarcted vs normal myocardium was attempted as early as 1986, using excised human hearts to distinguish infarcted from normal myocardium, and subsequently confirmed in several investigations.^37,41^ Moreover, studies have explored various methods to quantify image texture through numeric, statistical, and signal processing techniques such as fractals, Fourier transforms, and wavelet transformations.^37^ The recent development of novel computational approaches has enabled “radiomics” applications for broader adoption in clinical practice. Specifically, the present work extends our recent observations^7^ that specific analyzable trends in ultrasound texture information identified tissue-based changes in cardiac remodeling, and could predict patients who showed the presence of late gadolinium enhancement in myopathic hearts. In contrast to previous studies, we here show the feasibility of using ultrasomics features even from apical echocardiography views for developing ML models.

CNNs are effective for medical image analysis and have shown promising results with transfer learning from large ImageNet-trained models.^42,43^ We explored static and dynamic features, incorporating cardiac cycle variations with the use of radiomics and 3D-CNN.^44^ Dynamic models generally performed better than static models, but handcrafted ultrasomics outperformed 3D-CNN–based deep features in MI prediction. Handcrafted ultrasomics, therefore, may offer an advantage in capturing intricate tissue-level changes with smaller sample sizes than deep features extracted by 3D-CNNs, which typically require larger datasets because of their data-hungry nature.^45^

Among various clinical variables indicating prognostic significance in myocardial infarction, LS, measured by means of speckle-tracking echocardiography, is considered to be the most sensitive marker for identifying LV dysfunction.^46^ Deep learning–based techniques have recently helped reduce interobserver variability in assessing LS, regional wall motion score index, and LV ejection fraction.^47^ We therefore used a previously validated deep learning platform in our study^22^ to measure these clinical parameters and their interaction with the ML model for predicting MI. Interestingly, logistic regression analysis showed the value of ultrasomics in independently predicting MI even after adjusting for average LS and other echocardiographic parameters (Table 5). Ultrasomics provided significant incremental value over the averaged LS in distinguishing MI (Figure 3). Because regional or global LV dysfunction can arise from various causes, identifying MI as the underlying factor holds clinical significance for patients presenting with acute coronary syndrome. Our exploratory analysis (Supplemental Figure 4) also suggests that ultrasomics may be valuable in estimating total infarct size. These observations provide important preliminary evidence for future validation in larger patient cohorts.

For implementation, the proposed ultrasomics method may be integrated directly with existing ultrasound systems, offering automated cardiac image analysis. After the image identification steps, the software automatically performs segmentation, quality control, and radiomics feature extraction, requiring approximately 3–5 minutes per patient. Results can be displayed as visual overlays, ensuring transparency for straightforward interpretation. However, prospective studies would be necessary to study generalizability and potential implementation barriers in clinical settings.

There are several strengths of our study: the use of multicenter and open-source data to develop machine-learning models, validation using a core laboratory–assessed multi-institutional clinical trial database using CMR imaging as a criterion standard for infarct quantification, comparison with conventional echocardiography features, comparison with transfer learning–derived deep features and 3D-CNN models, use of harmonization techniques across multivendor echocardiography settings, and use of novel LOSO-CV for external validation. However, several limitations of this study also need to be acknowledged. First, the open-source dataset from HMC-QU did not include an apical 3-chamber view; however, the handcrafted radiomics model showed robust performance even without this additional view. Future studies need to include 3-chamber views, which may be relevant for accurate infarct size estimation. Second, the infarcted region is heterogeneous and consists of tissue characteristics ranging from necrotic to ischemic yet viable tissue and microvascular obstruction to intramyocardial hemorrhage. The differential effects of these features affect the future recovery of myocardial function and would need more in-depth analysis. Third, although vendor effects were statistically adjusted for, prospective implementation across varying image quality with an assessment of test-retest variability warrants careful investigation. Fourth, although our LOSO-CV framework improved generalizability with consistent performance (AUC: 0.84–0.94), future prospective multicenter studies with larger cohorts are needed to validate these findings further.

## CONCLUSIONS

The present data suggest that ultrasomics can enhance texture analysis in quantitative echocardiography by uncovering patterns imperceptible to the human eye. Specifically, this study investigated patient-level ML models and highlighted the potential of ultrasomics for myocardial tissue characterization in patients with acute MI. However, further validation in larger and more diverse cohorts—and direct comparisons with conventional echocardiographic parameters, such as LS—are necessary to confirm its generalizability and incremental clinical value.

## Supplementary Material

Supplement

## Figures and Tables

**TABLE 1:** Clinical Characteristics for the Cohort From Source A (N = 143)

	MI (n = 72)	Control (n = 71)	*P* Value

Age, y	65 (55–74)	60 (52–70)	0.15
Male	51 (71)	47 (66)	0.68
Diabetes mellitus	25 (35)	20 (28)	0.51
Hypertension	54 (75)	42 (59)	0.07
E, m/s	0.8 (0.6–0.9)	0.8 (0.6–0.9)	0.62
A, m/s	0.8 (0.6–0.9)	0.8 (0.7–0.9)	0.16
E/A	0.9 (0.8–1.2)	0.87 (0.7–1.1)	0.38
Average e′, cm/s	6.4 (5.4–7.8)	9.0 (8.0–11.3)	<0.0001
E/e′	11.6 (8.5–14.9)	7.6 (6.7–9.2)	<0.0001
LVEDV, mL	107 (83–132)	78 (70–102)	<0.0001
LVESV, mL	54 (39–78)	28 (24–37)	<0.0001
LVEF, %	48 (37.8–56.3)	63.3 (59.9–67.9)	<0.0001
LAVi, mL/m^2^	21.05 (17–25.9)	22.92 (18.1–28.2)	0.15
LVMi, g/m^2^	85 (74–99)	71 (63–81)	<0.0001
WMSI	2.1 (1.3–2.4)	1.1 (1.0–1.2)	<0.0001
LS, %	−10.3 (−13.8 to −7.8)	−17.3 (−18.9 to −15.7)	<0.0001

Values are median (Q1-Q3) or n (%), unless otherwise indicated. Continuous variables were compared with the use of a 2-sided Student’s *t*-test if normally distributed per Shapiro-Wilk test or Mann-Whitney *U*-test otherwise. Categoric variables were compared with the use of the chi-square test if all expected cell counts were ≥5 or Fisher test otherwise.

A = late diastolic transmitral flow velocity; E = early diastolic transmitral flow velocity; e′ = early diastolic relaxation velocity at the septal mitral annular position; LAVi = left atrial volume index; LS = longitudinal strain; LVEDV = left ventricular end-diastolic volume; LVEF = left ventricular ejection fraction; LVESV = left ventricular end-systolic volume; LVMi = left ventricular mass index; WMSI = wall motion score index.

**TABLE 2:** Clinical Characteristics for the Cohort From Source B (N = 129)

	MI (n = 38)	Control (n = 91)	*P* Value

Age, y	57 (51–67)	61 (51–68)	0.54
Male	29 (76)	54 (59)	0.10
Diabetes mellitus	6 (16)	17 (19)	0.89
Hypertension	18 (47)	52 (57)	0.41
E, m/s	0.7 (0.6–0.9)	0.7 (0.6–0.8)	0.41
A, m/s	0.7 (0.6–0.8)	0.7 (0.6–0.8)	0.85
E/A	1.1 (0.9–1.3)	1.0 (0.8–1.3)	0.41
Average e′, cm/s	6.6 (5.3–7.7)	7.6 (6.3–8.8)	0.008
E/e′	12.3 (7.9–16.1)	8.96 (7.3–10.8)	0.007
LVEDV, mL	96 (87–128)	96 (74–119)	0.43
LVESV, mL	45 (35–56)	30 (23–41)	<0.0001
LVEF, %	50 (40.5–52.8)	64.0 (59.0–66.0)	<0.0001
LAVi, mL/m^2^	27.4 (22.5–33.7)	32.0 (27.4–38.4)	0.01
LVMi, g/m2	91.6 (73.3–98.4)	76.9 (66.3–90.3)	0.08
WMSI	1.8 (1.2–2.1)	1.00 (1.0–1.0)	<0.0001
LS, %	−12.0 (−14.3 to −9.4)	−19.6 (−22.1 to −17.5)	<0.0001

Values are median (Q1-Q3) or n (%), unless otherwise indicated. Continuous variables were compared with the use of a 2-sided Student’s *t*-test if normally distributed per Shapiro-Wilk test or Mann-Whitney *U*-test otherwise. Categoric variables were compared with the use of the chi-square test if all expected cell counts were ≥5 or Fisher test otherwise.

Abbreviations as in Table 1.

**TABLE 3:** Clinical Characteristics for the Cohort From Source C (n = 250)

	Open-Source MIMIC-V-ECHO Database		Prospective Multivendor Database	
		
	MI (n = 32)	Older Control (n = 31)	Younger Control With Risk Factors (n = 111)	Younger Control Without Risk Factors (n = 76)

Age, y	71 (61–79)	82 (72–87)^a^	55 (42–66)	33 (24–43)^b^
Male	19 (59)	21 (68)	70 (63)	42 (55)
Diabetes mellitus	14 (44)	14 (45)	25 (23)	0 (0)^b^
Hypertension	10 (31)	31 (100)^a^	75 (68)	0 (0)^b^
E, m/s	0.8 (0.7–0.9)	0.8 (0.7–0.9)	0.8 (0.7–0.9)	0.8 (0.7–0.9)
A, m/s	0.7 (0.6–0.9)	1.00 (0.88–1.16)^a^	0.8 (0.6–0.9)	0.5 (0.4–0.6)^b^
E/A	1.1 (0.8–1.4)	0.7 (0.6–0.9)^a^	1.1 (0.8–1.3)	1.6 (1.3–1.8)^b^
Average e′, cm/s	7.4 (5.6–9.4)	7.0 (5.8–8.6)	9.6 (7.8–11.8)	13.2 (11.7–14.7)^b^
E/e′	9.3 (8.6– 12.5)	10.7 (9.1–13.6)	8.4 (6.7–9.7)	6.1 (5.4–7.1)^b^
LVEDV, mL	88 (69–99)	75 (58–99)	94 (76–116)	95 (78–113)
LVESV, mL	37 (25–52)	26 (19–32)^a^	35 (28–44)	38 (30–47)
LVEF, %	52.7 (48.3–65.2)	69.0 (64.0–71.9)^a^	63.0 (56.9–68.0)	61.7 (53.5–66.0)
LAVi, mL/m^2^	23.9 (20.3–30.8)	28.3 (20.6–33.8)	21.6 (17.9–24.9)	16.3 (14.3–20.4)^b^
LVMi, g/m2	86.3 (76.7–108.0)	76.0 (62.6–92.3)	65.8 (55.6–79.7)	61.6 (53.2–70.5)^b^
WMSI	1.0 (1.0–1.4)	1.0 (1.0–1.0)^a^	1.0 (1.0–1.0)	1.0 (1.0–1.0)^b^
LS, %	−14.8 (−17.1 – −12.0)	−17.8 (−19.4 to −16.3)^a^	−21.0 (−24.0 to −18.9)	−22.7 (−25.1 to −20.5)^b^

Values are median (Q1-Q3) or n (%). Continuous variables were compared with the use of a 2-sided Student’s *t*-test if normally distributed per Shapiro-Wilk test or Mann-Whitney *U*-test otherwise. Categoric variables were compared with the use of the chi-square test if all expected cell counts were ≥5 or Fisher test otherwise.

a *P* < 0.05 vs MI.

b *P* < 0.05 vs with risk factors.

Abbreviations as in Table 1.

**TABLE 4:** Performance of Machine Learning Models Developed Using a Leave-One-Source-Out Cross-Validation Approach

Modality	Source	Accuracy, %	Brier Score	Sensitivity, %	Specificity, %	F1-Score, %	AUC

HCR	Source A	75.5 (67.8–83.2)	0.17 (0.14–0.20)	91.7 (84.3–97.2)	59.2 (48.0–71.1)	79.0 (71.7–85.4)	0.84 (0.78–0.90)
	Source B	86.0 (80.6–92.2)	0.09 (0.06–0.12)	94.7 (86.1–100.0)	82.4 (74.4–90.4)	80.0 (71.1–88.6)	0.94 (0.90–0.97)
	Source C	80.6 (76.7–84.5)	0.17 (0.14–0.19)	70.7 (63.4–77.9)	85.3 (81.3–89.3)	70.1 (63.7–76.2)	0.85 (0.82–0.89)
	Overall	74.9 (71.9–78.1)	0.15 (0.14–0.17)	90.1 (86.4–93.7)	66.4 (62.3–70.7)	71.8 (67.9–75.7)	0.87 (0.84–0.89)
DTL	Source A	59.4 (51.7–67.8)	0.29 (0.25–0.34)	48.6 (37.3–60.6)	70.4 (59.7–80.3)	54.7 (44.4–64.3)	0.57 (0.48–0.67)
	Source B	71.3 (63.6–78.3)	0.22 (0.16–0.29)	84.2 (71.1–94.6)	65.9 (56.3–75.5)	63.4 (51.1–72.7)	0.80 (0.72–0.87)
	Source C	77.7 (73.5–81.6)	0.23 (0.20–0.27)	73.7 (66.4–80.8)	79.6 (75.2–84.2)	68.1 (61.7–73.8)	0.83 (0.79–0.87)
	Overall	68.6 (65.3–71.9)	0.24 (0.22–0.27)	63.4 (57.2–69.4)	71.4 (67.3–75.6)	58.9 (54.0–63.9)	0.74 (0.70–0.77)

Values in parentheses are 95% CIs computed using the bootstrapping technique. DTL = deep transfer learning; HCR = hand-crafted radiomics.

**TABLE 5:** Univariate and Multivariate Logistic Regression Analysis for Predicting MI Using Echocardiographic Variables

	Univariate Analysis		Multivariate Analysis	
		
	OR (95% CI)	*P* Value	OR (95% CI)	*P* Value

LVMi, g/m^2^	1.03 (1.02–1.04)	<0.0001	1.01 (0.99–1.04)	0.3648
LVESV, mL	1.05 (1.04–1.06)	<0.0001	0.99 (0.96–1.03)	0.6276
LVEF, %	0.88 (0.85–0.90)	<0.0001	0.94 (0.88–1.00)	0.0594
E/A ratio	0.86 (0.52–1.41)	0.5376	–	–
Average E/e′	1.21 (1.14–1.28)	<0.0001	1.11 (0.98–1.25)	0.1041
WMSI	31.91 (15.82–64.36)	<0.0001	0.48 (0.10–2.23)	0.3489
Averaged LS, %	1.71 (1.54–1.89)	<0.0001	1.52 (1.27–1.82)	<0.0001
ML probability, %	1.05 (1.04–1.06)	<0.0001	1.03 (1.01–1.05)	<0.0001

ML = machine learning; other abbreviations as in Table 1.

## PERSPECTIVES

### COMPETENCY IN MEDICAL KNOWLEDGE:

Cardiac ultrasound–based radiomics, also referred to as ultrasomics, extracts high-throughput pixel-level information and shows clinical feasibility for detecting infarcted myocardium.

### TRANSLATIONAL OUTLOOK:

Further development and validation of ultrasomics may provide CMR-level insights for myocardial tissue characterization and aid quantitative assessment of infarct size.

## ABBREVIATIONS AND ACRONYMS

a2c: apical 2-chamber
a4c: apical 4-chamber
CMR: cardiac magnetic resonance
CNN: convolutional neural network
DTL: deep transfer learning–based radiomics
HCR: handcrafted radiomics
LOSO-CV: leave-one-source-out cross-validation
LS: longitudinal strain
LV: left ventricular
MI: myocardial infarction
ML: machine learning
