# Supplementary material for: Ultrasonic Texture Analysis for Predicting Acute Myocardial Infarction
Source: JACC Cardiovasc Imaging. Author manuscript; Available in PMC 2025 Oct 25. (PMC12550873; doi:10.1016/j.jcmg.2025.06.018)
Supplement: Supplement [file NIHMS2115895-supplement-Supplement.docx]

# Supplemental Methods

## Image pre-processing attributes

**Supplemental Table 1** Image processing attributes from diverse databases.

| **Attributes** | **Source A: Retrospective trial (n=143)** | **Source B:**  **Prospective**  **Trial (n=129)** | | **Source C: Open-source and**  **multi-vendor**  **Databases (n=412)** | | |
| --- | --- | --- | --- | --- | --- | --- |
| Sites | RWJUH  (n=143) | DTU-STEMI  (n=40) | MSUH  (n=89) | HMC-QU  (n=162) | MIMIC-IV-ECHO (n=63) | WVUH  (187) |
| Timelines | January 2023 to July 2023 | August 2017 to September 2018 | August 2017 to September 2018 | 2018 to  2019 | 2017 to  2019 | August 2017 to September 2018 |
| Vendors | GE  (Vivid-9/95) | GE  (Vivid-9/95) | GE  (Vivid-9/95) | Phillips and  GE Vivid | GE  (Vivid-E90, E95, and S7) | GE (Vivid-9/95), and Hitachi (LISENDO 880) |
| Average frame rate of the converted DICOM | 30 | 25 | 50 | 25 | 31 | 30 |
| Average frames within a cardiac cycle | 13 | 13 | 23 | 17 | 13-15 | 13 |
| Spatial Resolution Post Segmentation for Ultrasomics | 1024x1024 pixels | 1024x1024 pixels | 1024x1024 pixels | 1024x1024  pixels | 1024x1024  pixels | 1024x1024  pixels |
| RWJUH = Robert Wood Johnson University Hospital, DTU-STEMI = Door-to-unload STEMI, MSUH = Mount Sinai University Hospital, HMC-QU = Hamad Medical College, Qatar University, MIMIC-IV-ECHO = Medical Information Mart for Intensive Care IV with Echocardiogram, WVUH = West Virginia University Hospital, GE = General Electric | | | | | | |

## Selection and Exclusion criteria for STEMI patients from RWJUH

STEMI was classified per the Joint ESC/ACCF/AHA/WHF Task Force.^1^ Briefly, this included ECG changes revealing 1) new ST-segment elevation in 2 contiguous leads with greater than 0.1 mV in all leads, with the exception of V2 or V3, 2) new ST-segment elevation in leads V2-V3 greater than 0.2 mV (men > 40 years old), 0.25 mV (men < 40 years old), or 0.15 mV (women), 3) Pre-existing left bundle branch block were further evaluated using the Sgarbossa's criteria.^2,3^ Exclusion criteria included (1) patients discharged to institutionalized care, (2) type 2-5 acute myocardial infarction (AMI), (3) co‐existing terminal illness such as cancer, (4) alternative diagnosis for elevated cardiac troponin values (e.g. myocarditis, pericarditis, non-ischemic cardiomyopathies, moderate-severe valvular heart disease), (5) pregnancy, and (6) technically insufficient imaging for 2 of the following 3 views: apical 4-chamber (A4C), apical 3-chamber (A3C), and 2-chamber (A2C).

## Handcrafted ultrasomics features

A total of 98 shape, 1^st^-order, and texture-based features were extracted for each of the 12 myocardial segments. Root mean square and spectral entropy were calculated for each feature across all frames in a cardiac cycle to capture temporal and spectral variations. Subsequently, all the ultrasomics features from a single segment from the four feature categories (98 from end-diastole, 98 from end-systole, 98 temporal, and 98 spectral) were compiled. Features from all 12 segments were gathered for each patient (totaling 2,352 features per view and 4,704 per patient) for feature engineering and machine learning model development.

## In the feature engineering process, the Boruta feature selection algorithm was employed to identify the most relevant and informative features for the predictive model.^4^ Boruta is a wrapper method built around a random forest classifier, designed to capture all features that are statistically significant with respect to the target variable. Using the BorutaPy implementation in Python, we iteratively compared the importance of actual features against randomized shadow features to determine their relevance. Features confirmed by Boruta as important were retained, ensuring that only those with strong and consistent predictive power were included in the refined training and testing datasets for model development.

1. ***Normalization of Ultrasomic Features***

We preprocessed the ultrasomics data using **standardization** to ensure that all features have a mean of 0 and a standard deviation of 1, bringing them to a comparable scale.^5^ This was done using the StandardScaler function from the **scikit-learn** library in Python. Standardization was applied by subtracting the mean and dividing by the standard deviation for each feature:

$$z=\frac{x-\mu}{\sigma}$$

Where 𝑥 is the original feature value, μ is the mean of the feature, and σ is the standard deviation of the feature. The StandardScaler method computes these values for each feature and transforms the data accordingly. This process ensures that each feature contributes equally to the analysis, preventing features with larger numerical ranges from dominating the model. It also accelerates the convergence of many machine-learning algorithms by improving their stability and performance. Additionally, it helps in reducing the effect of outliers, resulting in more robust model training.

## Harmonization of radiomics features

Radiomics features extracted from cardiac ultrasound images can vary significantly due to differences in imaging protocols and scanner settings across centers. This study acquired imaging data from multiple centers using various ultrasound systems, including GE Vingmed, Philips Medical Systems, and Hitachi. To address scanner-related variability and reduce batch effects in the radiomics data, we applied the ComBat harmonization technique—a widely used and robust method for correcting batch effects.^6^ ComBat was chosen for its ability to adjust both location (mean) and scale (variance) of feature distributions, making it particularly suitable for harmonizing radiomics data.

We followed a source-wise harmonization approach, where HCR and DTL features from each source were independently harmonized using a separate multivendor reference dataset not used for model training or evaluation (n=801); it served solely for harmonization. Radiomics features were available for all patients from either A2C or A4C views, with both views present in 73% of cases. For the rest, features from one view were sufficient. Median imputation was applied for missing values during ComBat harmonization. By aligning each source to this standard reference, ComBat effectively corrected scanner-induced variability, helping ensure consistency and comparability of features across different sources.

## Hyperparameters for the XGBoost model

**Supplemental Table 2** The hyperparameters of the patient-level XGBoost model.

| Parameter | Value |
| --- | --- |
| Number of trees | 50 |
| Maximum depth | 6 |
| Minimum child weight | 1 |
| Learning rate | 0.6 |
| eta | 0.3 |
| Sample rate | 1 |
| Subsample | 1 |
| Maximum bins | 256 |

## Comparing the performance of ML models utilizing DTL features from four 3D-CNN architectures

**Supplemental Table 3** Performance of ML models on the training dataset with cross-validation and test dataset, utilizing DTL features from cardiac cycle extracted from three 3D-CNN architectures.

| **Models** | **Groups** | **Accuracy**  **(%)** | **brier**  **score** | **Sensitivity**  **(%)** | **Specificity**  **(%)** | **F1-Score**  **(%)** | **AUC** |
| --- | --- | --- | --- | --- | --- | --- | --- |
| Channel-Separated CNN  (csn_r101) | Source A | 60.8 (52.4-69.2) | 0.43 (0.36-0.50) | 50.0 (38.2-62.0) | 71.8 (61.0-81.9) | 56.3 (45.7-66.2) | 0.59 (0.50-0.69) |
|  | Source B | 72.9 (65.1-80.6) | 0.23 (0.19-0.26) | 18.4 (6.9-32.4) | 95.6 (91.2-99.0) | 28.6 (12.0-45.3) | 0.49 (0.36-0.61) |
|  | Source C | 76.2 (71.8-80.3) | 0.24 (0.23-0.25) | 60.2 (51.4-68.1) | 83.9 (79.5-88.1) | 62.0 (54.5-68.9) | 0.78 (0.73-0.83) |
|  | Overall | 70.0 (66.5-73.7) | 0.28 (0.26-0.30) | 34.2 (28.4-40.5) | 89.8 (86.7-92.5) | 44.7 (38.1-51.1) | 0.56 (0.51-0.61) |
| Multiscale Vision Transformer  (MViT base 16x4) | Source A | 60.8 (53.1-69.2) | 0.30 (0.26-0.34) | 65.3 (54.2-76.1) | 56.3 (45.0-68.4) | 62.7 (53.8-71.0) | 0.60 (0.51-0.70) |
|  | Source B | 94.6 (90.7-98.4) | 0.13 (0.09-0.17) | 89.5 (78.8-97.5) | 96.7 (92.7-100.0) | 90.7 (83.3-96.8) | 0.97 (0.93-1.00) |
|  | Source C | 80.6 (76.7-84.2) | 0.15 (0.14-0.17) | 89.5 (83.5-94.3) | 76.3 (71.4-81.1) | 74.8 (69.3-79.8) | 0.89 (0.86-0.92) |
|  | Overall | 73.1 (69.9-76.3) | 0.18 (0.16-0.19) | 73.7 (67.9-79.3) | 72.8 (68.3-76.7) | 66.1 (61.4-70.4) | 0.77 (0.73-0.81) |
| Slow-fast ResNett50 model | Source A | 59.4 (51.7-67.8) | 0.29 (0.25-0.33) | 48.6 (37.3-59.5) | 70.4 (60.3-80.8) | 54.7 (43.7-64.5) | 0.57 (0.48-0.67) |
|  | Source B | 71.3 (62.8-78.3) | 0.22 (0.16-0.28) | 84.2 (71.4-95.1) | 65.9 (56.6-75.5) | 63.4 (51.1-73.2) | 0.80 (0.72-0.87) |
|  | Source C | 77.7 (73.5-81.6) | 0.24 (0.19-0.27) | 73.7 (66.2-81.3) | 79.6 (74.6-84.5) | 68.1 (61.8-74.2) | 0.83 (0.79-0.87) |
|  | Overall | 68.6 (64.9-71.9) | 63.4 (57.2-69.2) | 71.4 (67.0-75.7) | 58.9 (53.5-63.7) | 0.24 (0.22-0.27) | 0.74 (0.70-0.78) |

## Features of importance identified using pair-wise comparison discriminating infarcted and non-infarcted myocardium

**Supplemental Table 4** A pairwise comparison to identify the features that discriminate the infarcted segments against the non-infarcted segments within a view.

| Ultrasomic Features | p-value |
| --- | --- |
| NGTDM Strength at the ED frame | p<0.0001 |
| NGTDM Coarseness at ED frame | p<0.0001 |
| GLSZM Large Area High Gray Level Emphasis at ES frame | 0.00012 |
| NGTDM Strength at ES frame | 0.0002 |
| NGTDM Coarseness from spectral variations | 0.0005 |
| GLDM Gray Level Non-Uniformity from Temporal Variations | 0.0007 |
| NGTDM Coarseness from temporal variations | 0.0007 |
| GLDM Gray Level Non-Uniformity from ED frame | 0.0007 |
| NGTDM: Neighbouring Gray Tone Difference Matrix, GLSZM: Gray Level Size Zone Matrix, GLDM: Gray Level Dependence Matrix | |

## Effect of harmonization

**
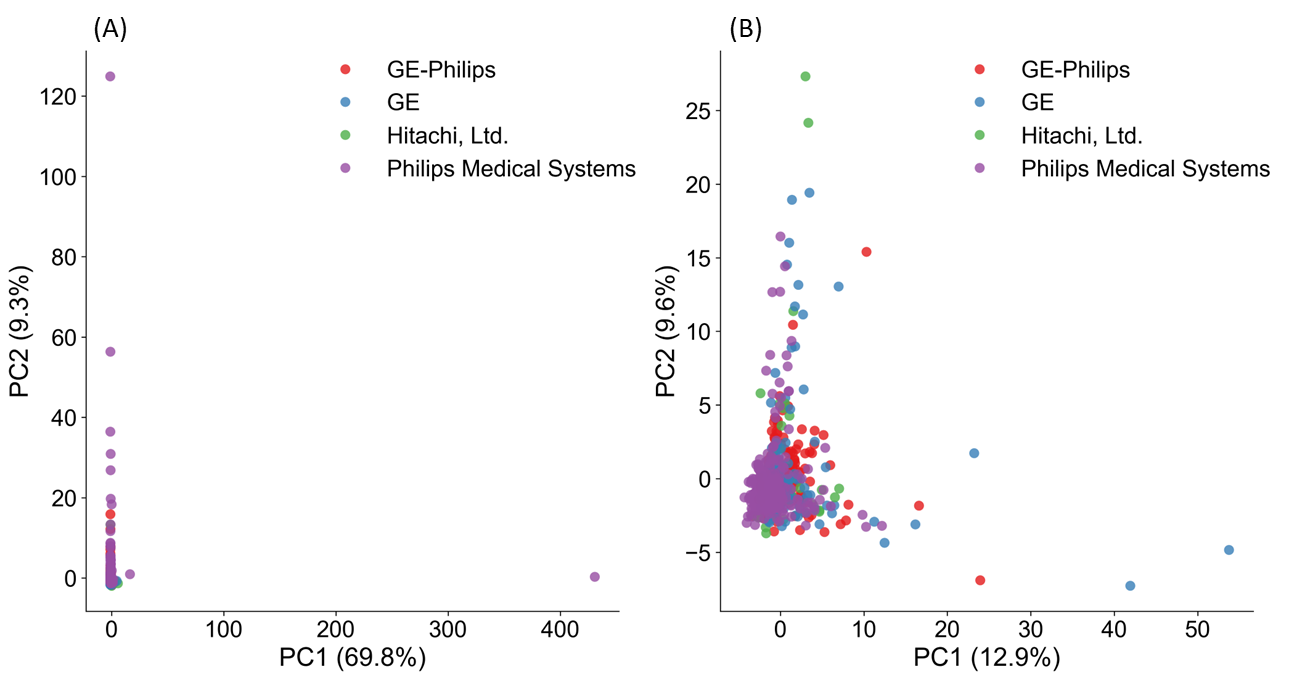
**

**Supplemental Figure 1**. Effect of ComBat harmonization on vendor-related variability in ultrasomics features. (A) PCA plots of all radiomics features, colored by vendor, demonstrate that prior to harmonization, features are strongly clustered by vendor, indicating significant batch effects. After ComBat harmonization (B), vendor-related separation is substantially reduced, and features from different vendors align more closely in the feature space. These results confirm that ComBat effectively mitigates scanner- and protocol-induced biases, allowing for improved generalizability in downstream analyses.


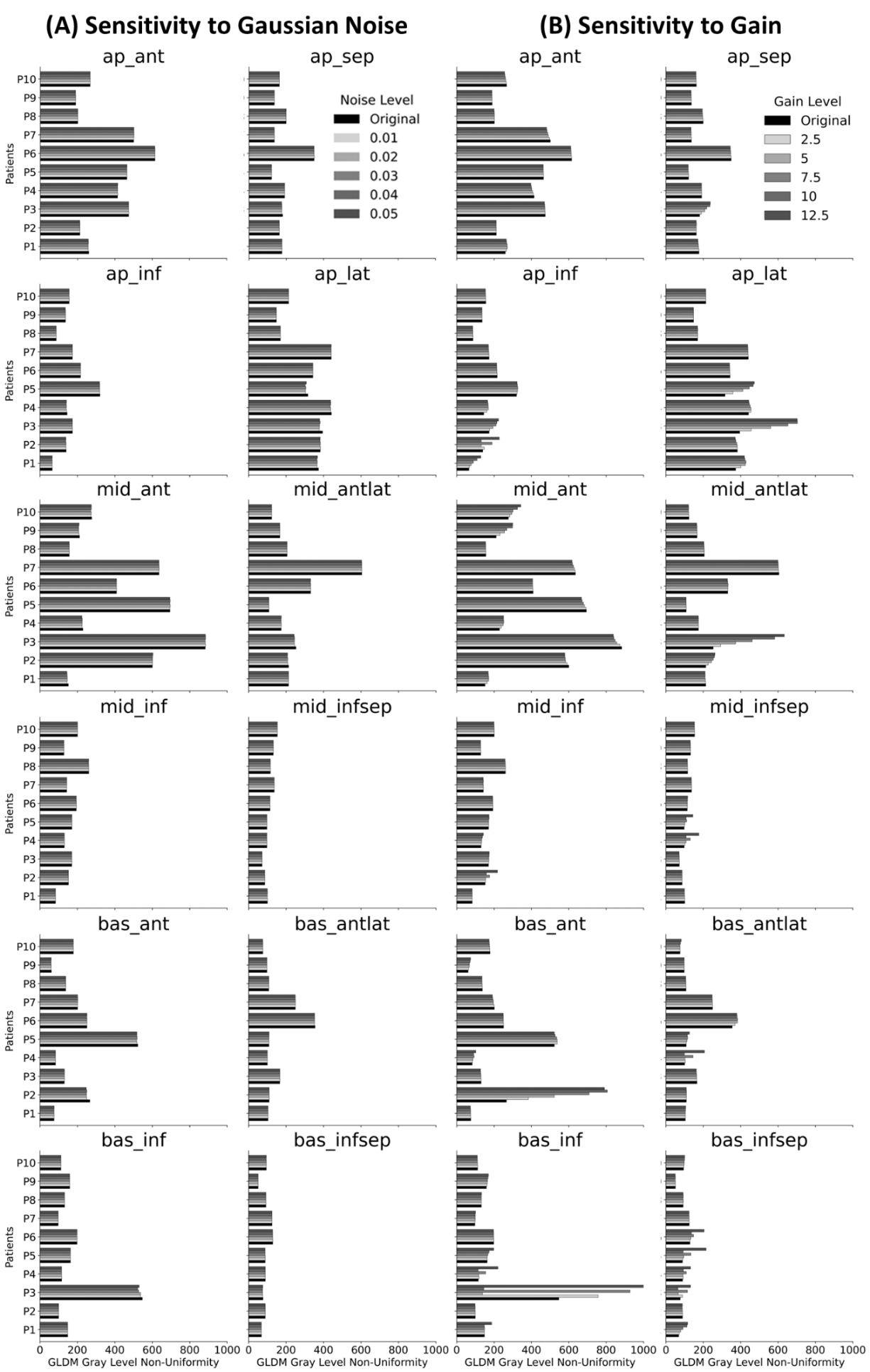


**Supplemental Figure 2** Impact of (A) Gaussian noise perturbations and (B) gain-level variations in image quality on ultrasomics features derived from static segmental images.


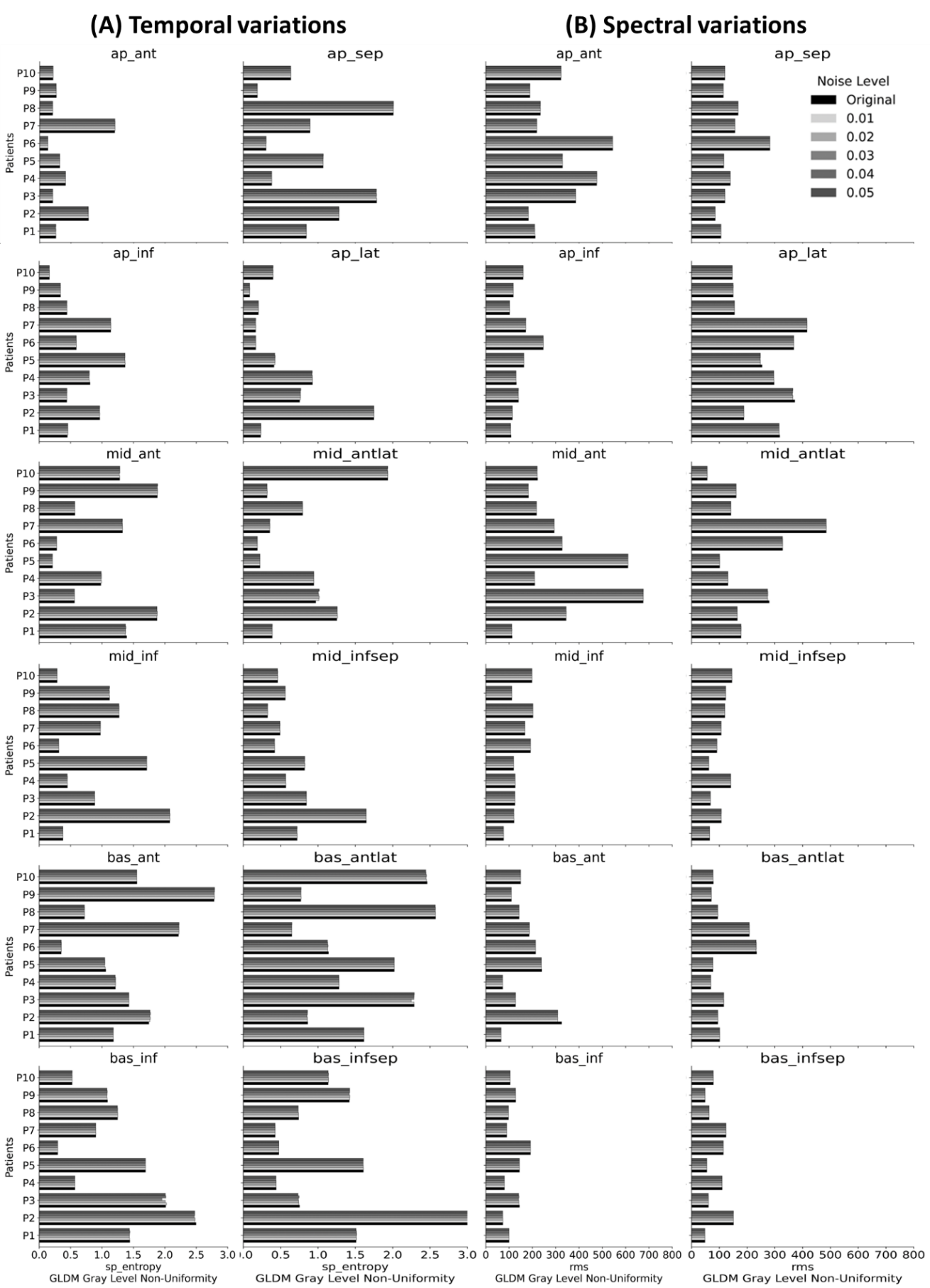


**Supplemental Figure 3** Effect of Gaussian noise perturbations in image quality on (A) temporal and (B) spectral variations in ultrasomics features derived from segmental images.

## Feasibility Analysis of ultrasomic ML model

**Supplemental Table 5** A pairwise comparison to identify the features that discriminate the infarcted segments against the non-infarcted segments within a view.

| **Models for Feasibility Analysis** | **Training Performance** | **% variations in training AUC** | **Test Performance** | **% variations in test AUC** |
| --- | --- | --- | --- | --- |
| Baseline Model | 0.89 | NA | 0.88 | NA |
| Model with 0.01 noise | 0.88 | 1.1 | 0.86 | 2.3 |
| Model with 0.03 noise | 0.84 | 4.5 | 0.85 | 1.2 |
| Model with 0.05 noise | 0.85 | 1.2 | 0.83 | 2.4 |
| **Average performance** | **0.86±0.02** | **2.3±2.0** | **0.85±0.02** | **1.9±0.7** |

## Prediction of infarct size using ultrasomics

We performed a predictive analysis using segmental ultrasomics features from both a2c and a4c views, selecting eight key features through recursive feature elimination. The resulting regression model strongly predicted CMR-derived infarct size, showing a robust association with an R² of 0.79 (95% CI: 0.66–0.86, **Supplemental Figure 3**). The correlation between ML probability and longitudinal strain was 0.37 (p = 0.025), and with WMSI, it was 0.28 (p = 0.096). While these results are promising, we need further validation and have acknowledged them in the limitation section.


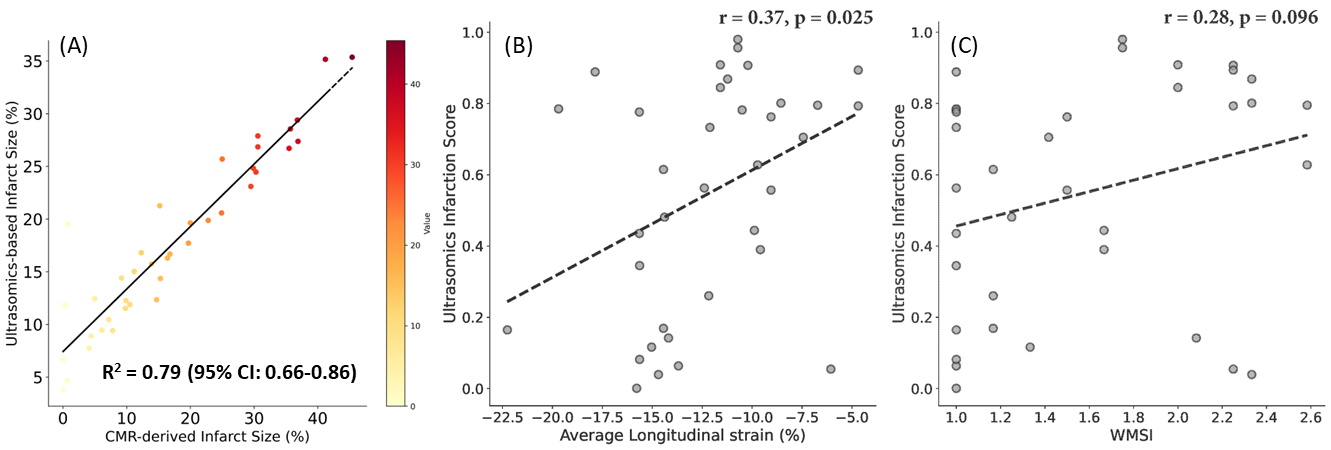


**Supplemental Figure 4** (A) Prediction of infarct size based on ultrasomics and its comparison with CMR-derived infarct size, (B) association between ultrasomics score and average longitudinal strain, and (C) association with WMSI.

**Supplemental References**

1. Thygesen K, Alpert JS, Jaffe AS et al. Third universal definition of myocardial infarction. *Eur Heart J.* 2012;33:2551-67.

2. Akbar H, Foth C, Kahloon RA, Mountfort S. Acute ST-Elevation Myocardial Infarction. StatPearls. Treasure Island (FL) ineligible companies. Disclosure: Christopher Foth declares no relevant financial relationships with ineligible companies. Disclosure: Rehan Kahloon declares no relevant financial relationships with ineligible companies. Disclosure: Steven Mountfort declares no relevant financial relationships with ineligible companies., 2024.

3. Smith SW, Dodd KW, Henry TD, Dvorak DM, Pearce LA. Diagnosis of ST-elevation myocardial infarction in the presence of left bundle branch block with the ST-elevation to S-wave ratio in a modified Sgarbossa rule. *Ann Emerg Med.* 2012;60:766-76.

4. Kursa MB, Rudnicki WR. Feature selection with the Boruta package. *Journal of statistical software.* 2010;36:1-13.

5. Bishop CM, Nasrabadi NM. *Pattern recognition and machine learning*: Springer, 2006.

6. Johnson WE, Li C, Rabinovic A. Adjusting batch effects in microarray expression data using empirical Bayes methods. *Biostatistics.* 2007;8:118-127.
